# What People Really Think About Safety around Horses: The Relationship between Risk Perception, Values and Safety Behaviours

**DOI:** 10.3390/ani10122222

**Published:** 2020-11-26

**Authors:** Meredith Chapman, Matthew Thomas, Kirrilly Thompson

**Affiliations:** 1 The Appleton Institute, Central Queensland University, 44 Greenhill Road, Wayville, SA 5034, Australia; matthew.thomas@cqu.edu.au; 2 Safety in Focus, PO Box 711, Narrabri, NSW 2390, Australia; 3 UniSA Business, University of South Australia, 101 Currie Street, Adelaide, SA 5001, Australia; Kirrilly.thompson@unisa.edu.au

**Keywords:** horses, risk, perception, safety, human–horse interaction, equestrian training-coaching, WHS

## Abstract

**Simple Summary:**

Equestrians continue to debate what they think, believe, feel and value as superior safety-first principles during human–horse interactions. Some of these varying opinions about how dangerous horses really are; the risk and what actions should be taken to minimise risk, appear to be determined by many different factors. This paper explored what humans say about safety around horses and identified what they perceived as important or less important to stay safe. We examined what elements influence human risk perceptions and behaviours (both positive and negative) during human–horse interactions. Some human safety choices were influenced by financial gain, level of experience, exposure to safety training and disregard for potential human injury as a deterrent for any safety change behavior. A significant percentage of participants accepted risk of human harm around horses with some choosing to take risks for sport achievements and others willing to place their horse’s safety above their own. This paper highlights benefits for the equestrian industry (work or non-work environments) in adopting some safety-first principles and standards, adopted by many high-risk workplaces such as safety training, risk assessment (horse-rider), improved communications and adequate supervision. Moreover, if the equestrian industry chose to implement these tried-and-tested safety principles, this would assist in mitigating risk and potentially reduce human horse-related injury and fatalities.

**Abstract:**

The equestrian industry reports high rates of serious injuries, illness and fatalities when compared to other high-risk sports and work environments. To address these ongoing safety concerns, a greater understanding of the relationship between human risk perception, values and safety behaviours is required. This paper presents results from an international survey that explored relationships between a respondents’ willingness to take risk during daily activities along with, their perceptions of risk and behaviours during horse-related interactions. Respondents’ comments around risk management principles and safety-first inspirations were also analysed. We examined what humans think about hazardous situations or activities and how they managed risk with suitable controls. Analysis identified three important findings. First, safe behaviours around horses were associated with safety training (formal and/or informal). Second, unsafe behaviours around horses were associated with higher levels of equestrian experience as well as income from horse-related work. Finally, findings revealed a general acceptance of danger and imminent injury during horse interactions. This may explain why some respondents de-emphasised or ‘talked-down’ the importance of safety-first principles. In this paper we predominantly reported quantitative findings of respondents self-reported safety behaviours, general and horse-related risk perceptions despite injury or illness. We discussed the benefits of improved safety-first principles like training, risk assessments, rider-horse match with enriched safety communications to enhance risk-mitigation during human–horse interactions.

## 1. Introduction

Horses can be dangerous, especially when humans ignore harmful situations or activities and choose not to control risk [1,2,3]. Moreover, horses have been identified as a hazardous animal [4,5,6,7], with horse-riding ranked as one of the most dangerous sports due to high accident frequency rates and the severity of human injuries [8,9].

For centuries horses have worked for humans, carrying them to war, ploughing their fields and providing a means of transportation [10,11,12,13,14]. The human–horse relationship has now extended from a basic human-survival relationship, to one that uses horses for human social activities, therapy, sporting and as a means of financial income [15,16].

However, humans continue to be exposed to ongoing risks [17] during horse interactions. The concept of risk exposure and potential harm during these interactions needs further investigation given humans generally present with varying levels of risk awareness. Regardless of previous research about the inherent dangers of horses and some behaviours that can cause serious injury or human fatality, humans will continue to domesticate equids (horses, mules and donkeys) for work or non-work purposes. Therefore, the inherent risk of human–horse interaction remains unchanged [18].

The horse is a sentient being (it perceives and feels); a large and fast animal (up to 500 kg, travelling up to 50 km/h); capable of harmful forces resulting in an unplanned human intercept (kick, bite or strike) whilst being agile [19,20,21]. Consequently, a horse is an imminent threat to human safety. In comparison, humans will never equal a horse’s physiological characteristics or capabilities [21] whereby, remaining the weaker or more threatened species during any interactions.

Previous studies of human–horse interactions have predominantly focused on injury and fatality statistics [17,22,23,24,25]. A few studies have analysed safety interventions highlighting the need for more robust preventative safety actions and risk mitigation opportunities. For example, air jackets reducing injury risk in falls [26], helmet usage for enhanced head protection [27], improved safety actions and behavioural awareness [23,28,29,30].

More recent research suggests we need to consider what factors contribute to different human behaviours and the risks they are prepared to take when interacting with horses [17,31,32,33,34]. This can be achieved by asking questions about what determines and possibly influences individual safety choices during human–horse interactions and more. There is a need to survey the influences of equestrian tradition, training, cultures, family and social constructs as this may also shed some light on why humans behave differently around horses [7]. By encouraging equestrians to speak out about their understanding of safety and what influences their decisions, will provide us with more insight into some of the elements that motivate human behaviours (unsafe acts and errors) around horses.

Equestrian has no formal regulatory framework for standardised safety and risk mitigation unlike high-risk workplaces, despite horses having a title of plant or equipment in work environments. However, some horse-related social, sporting and leisure associations, clubs and breed societies have attempted to include safety and risk mitigation processes to the best of their knowledge and expertise.

Unfortunately, many of these organisations are not for profit, supported by volunteers who are time-poor, but ‘love’ the sport. Many only contributing due to friends or family participation. This structure is unlikely to promote a consistent approach to safety and risk mitigation for both horses and humans, whereby some safety-first principles or activities may be done in the easiest, fastest or cheapest way, ignoring a correct procedure or rule to keep the horse-related activity going.

Whereas, some larger and funded organisations, like Equestrian Australia (EA), British Horse Society (BHS), and Pony Clubs and Racing do have reasonable safety and risk mitigation processes specific to their equestrian activity. Many have established rider-participant skill pathways (e.g., BHS, EA, Pony Clubs) [35,36,37,38] with Australian Racing having nationally accredited training programs (e.g., Certificate IV in Racing (Jockey-RGR40208) [39]. Some organisations provide training for equestrian officials (judges, course builders etc.), whereas others rely on volunteer support. A few associations offer coach programs, but not all. The majority of equestrian safety measures focus on low-level risk treatment (e.g., personal protective equipment) and some reporting. Little attention is given to environmental exposures, human-behavioural factors and risk assessment due to a general belief that riding is dangerous and humans can get seriously injured. 

In comparison, high-risk workplaces (e.g., mining and construction) do not operate or receive industry approval where applicable, without a strategically planned [40], tested and frequently reviewed safety management system [41]. With regular training to skill workers, safe work procedures, risk-assessment tools, safety-data analysis and other safety innovations, high-risk industries have minimised human risk exposures and promoted a safety-first culture [42,43].

We already know human behaviours and actions have complex relationships with values, attitudes and perceptions [44,45]. Therefore, to develop improved equestrian safety culture, it’s imperative we hear what equestrians feel and believe about safer human–horse interactions, before we can begin to influence and support change [46].

To complement the comprehensive body of work on human safety behaviours and actions, this study considers the values, beliefs equestrians place on safety-first principles and their perceptions of risk when interacting with a horse in a work or non-work environment. Furthermore, we examine relationships between the cohort of various countries, equestrian disciplines, their age, and gender and training exposures. Finally, this survey requests a self-analysis of each respondent’s level of horse-related experience and capabilities, whilst exploring perceptual differences between activities of daily living and horse-related sport risk.

## 2. Method

### 2.1. Measures

This study followed a self-report survey design. The survey contained a total of 48 questions, of which 34 were closed-ended or Likert and 14 were open ended. The survey consisted of five sections designed to provide data around the following:

#### 2.1.1. Horse Interests, Capabilities and Demographics

Respondents were asked to provide their age, gender and country they currently reside, including post or zip code. Respondents were also able to record their area of horse interest or discipline (qualitative response). Using yes or no responses respondents were able to distinguish if they classified their horse-related interest as work (income generating) or non-work (social, sport, leisure). In addition, yes or no responses classified training types when handling or riding horses. Numerical data were collected about hours per week and years of human–horse exposure times. 

Universally, equestrian does not define or outline a consistent approach [47,48] to riding or horse-handling capabilities, levels or skills due to the complexity of the horse-rider relationship [49,50]. Many equestrian organisation’s develop their own riding levels or they modify traditional categories from internationally recognised identifies such as the British Horse Society, Spanish or German Riding Schools and Federation Equestre Internationale [51]. Respondents were requested to self-declare their level of horse riding-handling capabilities using a 5-level rating scale (with definitions). This simple rating scale was adjusted from the British Horse Society Equine Excellence Pathways 1–4 stages in caring for and riding horses out. An easy one-word title was applied to the modified levels to provide more clarity and a basic definition [36]. The final self-assessment titles in this survey included:

(a) Beginner: needs supervision at all times when riding or handling a low-risk horse;
(b) Novice: able to ride out of an arena or yard independently and do low-level competition;
(c) Intermediate: confident to ride a variety of horses, basic training of a young horse with some supervision, and compete;
(d) Advanced: able to confidently ride and handle a variety of horses, able to ride a green- broke-young horse, and compete at moderate to higher level of competition; and
(e) Proficient: able to do all advanced rider skills, competently train others, and compete consistently in high level competition. (This category was added to include riders who have professional skills, earn an income or compete at the highest level of accomplishment within their equestrian interest).

#### 2.1.2. General Risk Perception

This was assessed using the standardized German Socio-Economic Panel (SOEP) survey [52] to measure respondent’s general willingness to take risks and their attitudes when car driving, financial matters, sports-leisure, health and career. Respondents rated their risk appetite using an ordinal measure on a scale from 0 (risk averse) to 10 (fully prepared to take risks). For the purpose of this survey the median was determined as 5. The SOEP survey design as applied to the German population identified heterogeneity and determinants of risk attitude in the population.

#### 2.1.3. Horse-Related Risk Perception

Various questions were constructed in this section of the survey to identify if humans viewed a horse as being dangerous (a hazard), having the potential to cause them harm and whether or not they had the ability to manage (control) this exposure [31]. Respondents’ opinions were questioned about their risk values and beliefs that may affect others [53,54], the value they place on human safety vs horse safety (where a choice is required) and their level of rule-orientation commitment to follow safety first principles. The aim was to measure each respondent’s tendency to take risks when interacting with horses. A Likert scale tabling six response types was used to record respondent’s risk attitudes, being rated from strongly disagree to strongly agree. This scale (six response options) was used to discourage ‘fence-sitters’ (indecisive respondents) whereby the respondent had to choose an answer that trended towards a risk aversion or risk-taking behaviour for each question, whilst promoting a controlled variance of opinion.

#### 2.1.4. Safety with Horses

Safety-first principles are traditionally adopted or mandated in workplaces along with, some social and sporting events. This section of the survey was designed to test respondents’ safety knowledge, safety first and risk management applications when interacting with horses. Again, a six-point Likert scale was used, accompanied by yes–no questions and an open-ended qualitative response section for respondents to list safety checks when approaching and mounting (before riding) a horse.

To determine what safety principles respondents would use ‘when a horse is displaying unsafe (at risk) behaviours’ to manage this exposure, a ‘hierarchy’ of control measures from 1 (most likely) to 6 (least likely) was tabled for respondents to choose which they would adopt to mitigate (reduce) the risk. The ‘hierarchy’ of control options used in this survey was adopted from the National Institute for Occupational Safety and Health (NIOSH) prevention through design program to prevent or reduce occupational injuries, illnesses, and fatalities [55,56,57].

#### 2.1.5. Injury-Illness During Horse-interaction

Following on with the perception that horses are dangerous, this section of the survey was designed to provide quantitative data regarding injury, illness, and fall results. This survey section also identified human permanent incapacity and how this interrupted a return to normal daily activities. All of these questions required a nominal value or a yes-no answer. Other questions in this section referred to safety with horses, taking more precautions following a horse-related injury or illness and the reporting of these incidents and near miss (nearly being harmed) events.

### 2.2. Procedure

Survey respondents were invited to participate via social media primarily Facebook, however several articles in horse journals (equestrian publications) and conference presentations were completed prior to the survey being active on-line. In addition, due to the diversity of equine disciplines and the extensive geographical contacts known by the researchers, dissemination of the survey was distributed to work-related, sporting, media and other social contacts.

The survey was available from 25 April 2018 to 28 August 2018. Of the 1718 starters, 1273 completed the entire survey and only these results were used for data analysis. This is consistent with a low attrition (drop out) rate of 22% [58]. The criteria for inclusion was being a minimum of 14 years old and having an association with horses for work or non-work purposes. This research was conducted in accordance with CQU Human Research Ethics Committee protocol number H17/02-026.

### 2.3. Data Analysis

Both qualitative and quantitative data were collected using the on-line tool survey-monkey (https://www.surveymonkey.com/r/HumanRiskPerceptionSafetyandHorses). The majority of the 48 questions reported quantitative responses, with only eight being qualitative. Initially all responses were collected using a computerized Statistical Package for Social Science (SPSS) V25. SPSS facilitated the exporting of all data into an excel spreadsheet for cleaning purposes prior to data analysis.

Original data were saved and a new working excel spreadsheet was created for the purpose of data cleaning. Each column within the spreadsheet was given a title to identify corresponding survey questions and to sort quantitative from qualitative responses. Quantitative data included yes–no questions, nominal values and ordinal numbers (1–6) to describe various questions requiring a value ranging from most likely to less likely.

Quantitative outliers were identified and adjusted by winsorising [59]. All other data were inspected, with missing values replaced, corrected text to nominal values and data that were inaccurate being discarded. Finally, comparative SPPS data analysis was determined using descriptive and inferential statistics, ANOVA, frequency and cross-tabulations. For ANOVA analyses, the factor variables were grouped according to natural categories (gender) or binned into equal groups (low, medium and high). Subsequent post hoc analyses between groups were undertaken through comparison of means using Bonferroni adjustment for multiple comparisons. This paper only reports findings from quantitative data analysis and the value respondents place on safety-first principles and risk perceptions when interacting with a horse in a work and non-work environment.

Qualitative data were also collected that included a few questions about safety processes when handling or riding horses. In addition, data about respondents’ country of origin, horse discipline or equestrian interest, preferred training methods, frequency and overall general comments were identified. Many respondents documented detailed comments, concerns and feelings about this research. Qualitative results will be presented elsewhere.

### 2.4. Ethics Statement

Ethics approval was received by Central Queensland University Ethics Committee. Approval number H17/02-026 / 0000020510 initial date 28 March 2017.

## 3. Results

### 3.1. Respondents

The survey was attempted by 1718 respondents, with a total completion rate of all questions being 1273 equestrians with representation from 25 different countries. The majority of respondents who named their country of origin, were from Australia (including New Zealand). The United States of America and United Kingdom (grouped as England, Scotland, Wales and Ireland) had moderate representation. The remainder of the respondents were one-off geographically scattered countries such as, Egypt, Canada, France, Iceland, Hong Kong, Poland, Zimbabwe and more.

**Figure 1:**
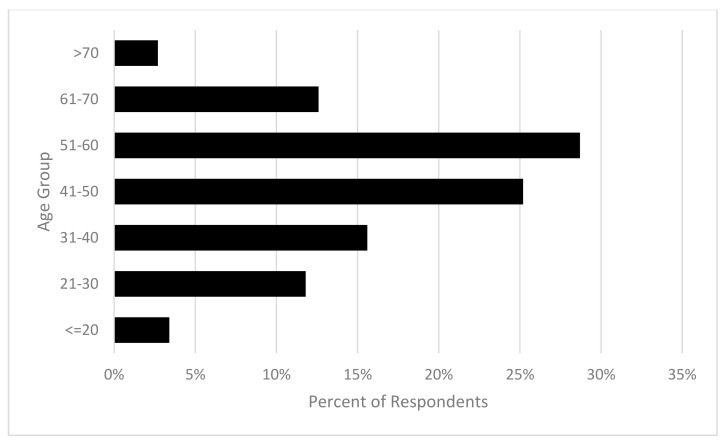
Distribution of respondents’ age

**Figure 2:**
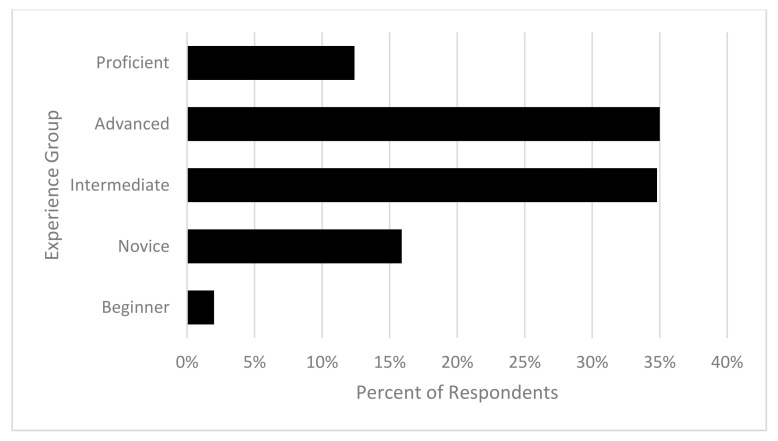
Distribution of respondents’ self-rated experience.

The survey sample was predominantly female (90.2%; *n* = 1148), with only (9.8%, *n* = 125) males and an overall mean age of the sample being 46.99 (SD = 13.71, range = 15–80) years. Some respondents reported earning an income from horse-related activities (29.3%; *n* = 373), with the majority participating in leisure or sporting (70.7%; *n* = 898). The majority of respondents (69.8%; *n* = 888) self-rated their level of experience with horses as intermediate or advanced (see Figure 1 and Figure 2).

### 3.2. Self-Report Safety Behaviours

**Figure 3:**
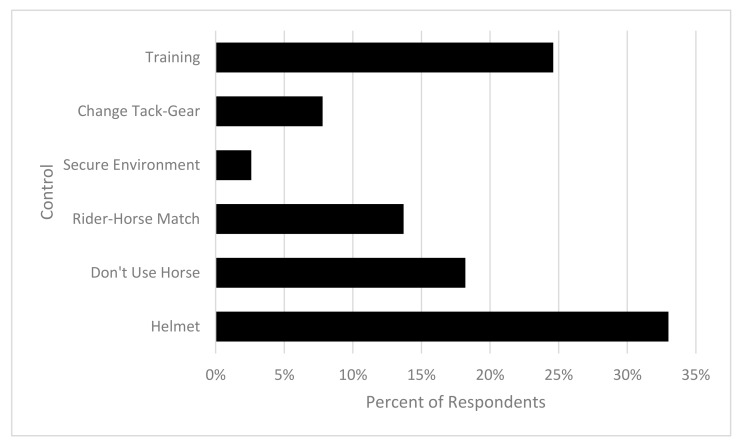
Distribution of highest ranked controls.

Respondents were asked to rate their safety actions in order of priority (No. 1 most to No. 6 least likely) if a horse was displaying unsafe behaviours. The most likely response was to wear a helmet (33.3%; *n* = 435). The next highest response was to no longer use the horse for a selected activity (30.5%; *n* = 402), then identify a better horse–rider–handler match (30.1%; *n* = 397), followed by keeping the horse contained in a secure enclosure (28.2%; *n* = 372), change tack or gear (27.8%; *n* = 366). The least likely response was to provide more training, supervision and limit activities with the horse (27.3%; *n* = 360). Of the respondents (76.1%; *n* = 965) had received training in horse handling, (85.2%; *n* = 1081) had received training in horse riding, and (48.4%; *n* = 614) were currently undertaking professional training (see Figure 3).

Analysis of respondent safety beliefs, values and interests showed (10.7%; *n* = 136) believed they were unable to control risks around horses with (12.5%; *n* = 159) agreeing it was okay to drug a horse to make it safer to ride. The majority of respondents were prepared to follow safety rules, even when others were not (96.1%; *n* = 1223) and similarly (91.1%; *n* = 1159 agreed an experienced rider not using safety equipment may influence safety values and beliefs of less experienced riders. A few respondents (3.6%; *n* = 45) believed if a horse had a history of unsafe behaviours, it was still safe for an inexperienced rider.

Over a quarter of the respondents (25.7%; *n* = 327) were willing to put their horse’s safety before their own. Of the respondents, (81.1%; *n* = 1037) reported having at least one horse-related injury, with an average of 3.28 injuries (SD = 6.19) reported per respondent. Moreover, (74.2%; *n* = 944) reported they now take more safety precautions around horses, following an unsafe horse interaction or a serious injury-illness.

The maximum number of reported rider falls from a horse in 12 months was 20, with a mean of less than one during this time. However, less than half (46.8%; *n* = 595) would record a horse-related injury or illness if an on-line reporting data base was available and (89.2%; *n* = 1135) of the respondents currently did not report human horse-related near miss events.

### 3.3. General Risk Perception

The six dimensions of propensity towards general risk-taking were highly inter-correlated. Males exhibited a higher propensity towards risk taking in general (*F*(1,1271) = 12.984, *p* < 0.001) and across all specific dimensions of risk-taking except health-related risk taking. Younger people were more likely to relate to perceptions reflecting general risk-taking than older respondents (*F*(6,1255) = 3.694, *p* = 0.001) (see Table 1).

Those respondents who derived income from horse-related activities exhibited a higher propensity towards risk taking in general (*F*(1,1269) = 15.860, *p* < 0.001) and across the specific dimensions of risk-taking in sport (*F*(1,1269) = 9.834, *p* = 0.002) and in occupational settings (*F*(1,1269) = 36.934, *p* < 0.001).

There was a significant relationship found between respondents with higher numbers of injuries (upper tertile injury rates) and an increased propensity towards risk-taking compared to respondents with lower injury rates, in terms of the specific dimensions of risk-taking in occupational settings (*F*(2,1275) = 4.125, *p* = 0.016) and health-related risk taking (*F*(2,1275) = 3.684, *p* = 0.025), but not sport (ns).

### 3.4. Horse-Related Risk Perception

There were no consistent relationships between the demographic variables of age or gender and horse-related risk perception. There was no relationship observed between whether a person had received a horse-related injury and their perception of horse related risk. However, respondents with higher numbers of injuries (upper tertile injury rates) were significantly more likely to agree with the statement that all horses can cause harm (χ2 (2,1278) = 6.268, *p* < 0.05).

There were no relationships observed between propensity towards risk taking in general and perception of a horse as a hazard or that all horses could cause harm. However, a higher propensity towards risk-taking in sport was associated with disagreement with the statements that a horse is a safety hazard (*F*(1,1545) = 4.961, *p* = 0.026) and that all horses could cause harm (*F*(1,1545) = 9.643, *p* = 0.002).

### 3.5. Horse-Related Safety Behaviours

Findings demonstrated that equestrian experience and income generation were associated with unsafe behaviours, whereas training was associated with positive safety behaviours.

There was a statistically significant relationship found between respondents’ reported engagement in horse-related safety behaviours and reports of experience as advanced to proficient. In particular, these respondents were less likely to follow rules (χ2 (2,1273) = 9.824, *p* < 0.05) with an increased propensity towards not wearing a safety helmet at home (χ2 (2,1273) = 46.909, *p* < 0.001) or in a work environment (χ2 (2,1273) = 41.348, *p* < 0.001).

Respondents who derived an income from horse-related activities had an increased propensity to compromise their safety before that of their horse (χ2 (1,1271) = 6.546, *p* < 0.05), not wear a helmet when riding a horse at home (χ2 (1,1271) = 16.045, *p* < 0.001), and ride largely in a work or competitive context (χ2 (1,1271) = 8.226, *p* < 0.01).

There was a statistically significant relationship found between respondents who had completed some form of safety training (formal or informal) and positive horse-related safety behaviours. These respondents agreed that handling (χ2 (1,1268) = 6.445, *p* < 0.05) and riding (χ2 (1,1269) = 5.763, *p* < 0.05) horses had an increased likelihood of risk. Safety-trained respondents agreed they had more control of risk when riding horses (χ2 (1,1269) = 7.834, *p* < 0.01) and as trainers had an increased propensity to influence trainees (less experienced) safety values and beliefs.

Proactive safety behaviours like following rules when handling (χ2 (1,1268) = 12.423, *p* < 0.001) or riding (χ2 (1,1269) = 9.228, *p* < 0.05) horses, with higher risk awareness by wearing a helmet handling horses at home (χ2 (1,1268) = 10.853, p < 0.001) or during work or competition (χ2 (1,1268) = 5.405, *p* < 0.05) was exhibited by those with training having a higher propensity towards safety-first principles.

## 4. Discussion

### 4.1. General Risk

The mean age of female respondents in this survey was 45 years and we were unable to identify a substantial finding between age, gender and horse-related risk. This study supports previous research of young males being more likely to take ‘general risks’ [60,61], despite the small survey sample of equestrian males with a mean age of 50 years. Horse-related injuries is well researched identifying young inexperienced (amateur) female riders under or around 20 years of age are the at-risk-group for accidents and fatalities [62,63,64].

The respondent sample may be considered typical in risk outcomes as income (money) [65] typically accelerated some human risk-taking opportunities in general sport, occupational settings and during some horse-related activities. This finding may indicate that humans are more likely to abandon safety-first principles and prepared to put themselves or others at a higher risk of injury or death, to obtain financial gain. More research in needed to identify the higher value humans place on income compared to their own life, their risk perceptions and willingness to accept this risk [66].

### 4.2. Horse-Related Risk Perception

#### 4.2.1. Acceptance of Risk, an Inherent Part of Interacting with Horses

This study demonstrated 36.2% (*n* = 460) of the respondents were fully prepared to take some risks in general activates of daily living. Acceptance of risk in occupations (27.5%; *n* = 350) and during sport leisure activities (40.5%; *n* = 515) may explain why some humans accept the risk of being injured around horses. Deroche et al. identified following a study of psychological mediators in sport injury, that the perception of lower levels of risk were associated with a higher risk of injury [67]. Many respondents still reported choosing to interact with horses knowing they are dangerous, with an acceptance that getting hurt is an inherent part of human–horse interactions in both sport and work (income) environments.

Risk acceptance means a human may participate in an activity knowing the risks with or without putting any controls in place. A study by Young et al. interviewed 16 Canadian athletes (e.g., ice hockey, football and rugby) to explore their personal acceptance of injury in various sports. In some cases it was expressed by the athletes as being masculine and somewhat highly valued living with ‘everyday pain’, sporting violence and even death [68]. Others may choose not to mitigate risks when engaging in a potentially dangerous situation (sport). Moreover, accepting it is unsafe, normalising the risk [69] or engaging sport as a dependency or addiction [70]. 10% (*n* = 127) of the respondents in the survey believed they could do nothing to mitigate horse-related risk. Therefore, a belief has already been formed about horse-related risk characteristics and its severity. This belief is unlikely to change without further interventions, such as risk analysis, safety training and the sharing ideas or experiences to be safer around horses [71]. Other sporting activities like running reported similar beliefs, being guided by the runner’s behavioral factors and lack of respect for physiological limitations and injury prevention [70].

A few behaviourists have tried to explain why humans perceive risk differently, again giving some insight into why some safe and unsafe outcomes occur during human–horse interactions. Myers suggests it’s a common sense approach, educated guess or intuition (heuristics), a mental (cognitive) shortcut to reduce decision making [72]. For example, seafarers will report their experience and knowledge is entrenched in a common sense method of seamanship. Thus, the use of documented safety procedures is overkill [73]. Myers theory and explanation may lead to inaccurate, unsafe or biased safety decisions. For equestrians an example can be putting a helmet on (short-cut solution) rather than spending more time on training or selecting a more suitable horse for a task or sporting activity.

In comparison, social or cultural (anthropology) influences [74] may also explain equestrian thinking, whereby choosing to belong to a specific horse-related discipline and adopt or conform to what is deemed acceptable safety practices within this group [75]. Similar findings were highlighted in a study of German Olympian athletes, whereby the elite group had a positive correlation to risk acceptance both physical and social within their sport environment [70]. Finally, Kasperson et al.’s theory of social amplification of risk (interdisciplinary approach) includes human psychology, social and cultural perspectives [34]. This theory is influenced by public opinion through the increase of an acceptable response to a single risk or risk event [76]. Kasperson et al.’s theory is also captured in a review by Nixon of some American sport articles that examined why athletes accepted the risk of pain and injury in sport. The findings suggest athletes identify with a set of values and beliefs to accept risk and pain in sport, due to the nature and implications of culture and social messaging [77].

Further research is required to determine if apparent risk acceptance led to or was a result from engagement in more or less risk controls. In addition, to explore human risk-acceptance behaviours amidst pending danger, knowing how serious a fall from a horse or unplanned ground interaction can be [78]. This research may provide further insight into equestrian cultures, and human risk acceptance around horses.

#### 4.2.2. Risk Perception Variations and Influences

Many of the respondent’s thoughts and beliefs appeared to be embedded in a group-based philosophy. Humans tend to feel more comfortable and accepted in groups [79]. Equestrian activities (work or non-work) cluster around similar interests, ideas or associations, for example pony club, dressage, veterinaries, jumping and farriers. Different cultural groups usually share similar beliefs. This was verified by the data when similar equestrian groups reported like-minded risk-related responses or comments.

Regardless of a perceived personal risk or threat, responses are regularly sanctioned by group agreeance or opinions. Sticking together enables strong cultural-social bonds and provides a protective barrier for varying horse-discipline identities and differences of opinions [80,81]. Whereby, an individual’s beliefs, values and risk perceptions if opposing can be hidden within a united group-voice. Moreover, a human may be less likely to express a safety concern or belief for fear of not ‘fitting-in’ the group, being rejected or different, thus creating intellectual conflict and an unwillingness to speak-up or accept a change in tradition or culture.

The human need to belong, think alike, network and protect each other’s beliefs and behaviours, can be liked to many herd animal behaviours [82,83]. Some human behaviours likened to those of herd animals may unintentionally cloud independent judgment resulting in more exposure to imminent danger or a hazard. The concept of tribal-bonding, experiencing group thoughts [84] and aspirations was evident in this survey, with many respondents sharing similar equestrian activities, along with related safety beliefs [85].

#### 4.2.3. Willingness to Participate in Risky Activities

If it looks and feels good humans are often willing to take a risk, despite any known or unplanned negative consequences. This behaviour choice can be called ‘desired risk’ participation, usually observed during high-risk sports and work activities [86]. Research has found that those who participate in high-risk sports are predominantly extroverts with Type A personality characteristics and they tend to be emotionally stable [87,88]. Some equestrian sports perceived as higher-risk may include cross-country eventing, bull-fighting on horseback, campdrafting and rodeo bronc riding. Similarly, non-equestrian sports such as hang-gliding, scuba diving, rock climbing, mountaineers and skydiving can be defined as ‘extreme sports’. Athletes who participant in any high-risk sport are often anecdotally labelled as mad!

However, this alleged title is often accompanied by strong leadership skills, emotional stability, independence and low levels of anxiety [87]. Some respondents in this survey had an increased propensity to disassociate horse-related human risk and potential harm to achieve sporting accolades and recognition. Therefore, it is not unreasonable to conclude they may present with similar character traits, identified in athletes who participate in extreme sports.

In addition, the concept of sensation or stimulation seeking has been given some consideration by researchers [46,60,89] and this may explain why some humans take more risks around horse than others. This was particularly evident with respondents who did not believe a horse was hazardous and could cause them harm, being overlooked when income and success was a stimulus. Some athletes could be described as having a stimulus addiction, whereby daily activates of living may be somewhat routine and boring, so pushing their physical and emotional boundaries becomes exciting [87]. In these circumstances, feelings of human dread or fear in a dangerous situation are expelled from normal human thinking processes. Some equestrians may fit into the sensation-seeking group [90], oozing confidence, with high self-efficacy and a strong desire to master the challenge of any high-risk activity, to control oneself and their environment [87,91,92].

### 4.3. Horse-Related Safety Behaviours

#### 4.3.1. More Experience, More Safety Short-cuts

To assist respondents in self-rating their level of capability they were provided with a brief definition of an adjusted 5-level-skill-scale based on the British Horse Societies Pathways program. The skill definitions prompted each respondent to consider during handling or riding a horse:

(a) do they need supervision,
(b) what type of environment can they safety interact with a horse and maintain control,
(c) what type of horse (level of training-risk) can they interact with and finally,
(d) what level of competition in their selected discipline with their current capabilities could they achieve. It was the respondent’s subjective opinion of themselves to determine their own rating.

Respondents who self-rated their level of experience and horsemanship skill as advanced or proficient, appeared more willing to take risks around horses and not follow simple, tried and tested safety-first procedures. Recent research has highlighted the potential for participants in equestrian sports to overestimate their level of knowledge or skill [93]. Sometimes, we just do not know, what we do not know! David Dunning and Justin Kruger (professors of psychology), explored this theory in 1999 and they describe their findings as the Dunning–Kruger effect [94]. It is simply defined as a cognitive bias (blind-spot), whereby humans have a tendency to overestimate their knowledge or ability. This theory suggests that the most competent humans usually underestimate their level of capabilities [94].

Whereas, unskilled or less capable humans (not necessarily incompetent) overestimate their abilities [95]. In many circumstances humans are more likely to be misguided by their own choices, even if they believe they are right and justified. However, one way a human can determine if their judgments and choices are accurate is if they receive guidance, a signal, or engage a mentor (coach or supervisor). Therefore, the human with superior knowledge and skill is then able to inform the other (trainee or less-skilled) of their ‘truth’ being more right or wrong in their selected decision.

With an increased level of skill and knowledge on safety around horses, it would be fair to say that humans should take less risk, be more proactive, diligently follow safety processes and lead by example, encouraging others to follow. However, equestrian respondents in the survey who self-rated above intermediate skill level were more risk-tolerant.

This suggests the more confident humans become, especially with some risky sports or work environments, the more likely they are to take short-cuts even when they know the consequences can be serious or fatal [96]. Being over-confident can result in harm, also lead to complacency and human error [97]. For example, leaving a riding helmet off for one-ride, not checking gear (tack) for frayed and worn parts or skipping usual safety groundwork processes to fast-track training. Therefore, when a safety short-cut works and no harm occurs, humans often embellish their choices, ability, and skill-level. With this adherence to best safety practices diminish and more short-cuts are likely to occur. Equestrian researchers and industry should explore this finding further, given research validates less competent humans often exaggerate their level of skill and knowledge [98].

#### 4.3.2. Safety Trade-Offs for Income

Money (income) has a strong influence over human decision making, regardless of the outcome being good or bad. This study suggests respondents who received an income during human–horse interactions, chose to take more risks. This was particularly evident for those who self-rated high competency levels for horsemanship and riding skills. Safety trade-offs overshadowed by income, especially in competitive occupational and sporting environments, exposed a disregard for safety-first principles, despite the human knowing the harmful consequences [52,92].

Trade-offs for income regardless of the risk to human health or wellbeing seems to be accepted in other contexts like, sex workers [99,100], income per capita, with less preparation for natural disasters [101] and rising urbanisation escalating global risk of obesity and type 2 diabetes [102]. Research has recognised safety-first human behaviours involving a problematic task, may change if rules are not reinforced and money becomes an incentive [103]. Society may perceive horse-related activities or sports as expensive, only rich people being able to afford them and tough luck if accidents happen. However, this research has identified the people most at risk are those who are engaging in a high-risk activity to make a living. Further research in compromised (income driven) unsafe human behaviours or choices, may benefit the equestrian industry and humanity in general.

#### 4.3.3. Safety Includes the Use of Drugs

Drugs are used by humans for medical or mental health conditions, pleasure and social purposes, this being an accepted and well-known practice in society [104,105]. As a result of taking drugs humans can experiences changes to their state of mind, pain levels or disease along with or without contraindications usually explained by the administering or prescribing medical professional. In addition, it is well known that many forms of drugs and substances are used illegally an often over-used or abused by humans [106,107]. Drugs and substances are used by humans (some unqualified) for horses (prescribed or not by a veterinarian) for the purposes of performance enhancement, calming, horse-health, welfare or sedation of a horse [108,109,110,111].

There are many legitimate reasons to administer drugs or substances to horses, however some survey respondents believed that drugging a horse to make it safer to ride was okay. This belief raises a number of safety, legal and horse welfare concerns. (1) If a horse is unsafe to ride, masking or intermittently controlling an unwanted or unsafe behaviour with a drug or substance does not fix or change the initial problem behaviour, even though it may ‘take the edge off it’. (2) There is an opportunity to misuse the administration of drugs or substances. Such as, frequency and dosage administration for the size or breed of the horse, accessibility (responsible distribution and monitoring by supplier) and long-term health or other effects on the horse’s welfare. Finally, (3) if a horse receiving drugs to make it safer to ride changes hands (sale, lease or other), the opportunity for non-disclosure of previous usage by the owner is very likely.

#### 4.3.4. PPE, Best Form of Safety Control

Helmets still reign supreme over all other safety controls when humans interact with horses. Helmet usage is frequently a topic of debate amongst equestrians, horse-related disciplines, and other sports, with the perception that if the lid (helmet) is on your safe to ride [112]. Research supports the wearing of helmets as a safety tool [113,114,115,116,117] however, not all equestrians wear one.

The use of a helmet for some may suggest an element of ‘risk compensation’ whereby a human changes their behaviour becoming more careful when sensing increased risk, and with a helmet less careful if they feel protected. However, many horse handlers and riders continue to sustain serious head-body trauma and even die when wearing a helmet [118]. While personal protective equipment (PPE) should be part of human–horse risk management, it is rated as the ‘lowest’ and ‘least’ effective safety control for any ‘high-risk’ activity including human–horse interactions [17,119,120,121].

Effective risk management supports a reduction of human injury-illness and fatalities in both work and non-work environment [17,122]. Improved safety controls usually require more planning and implementation than PPE. Some more effective (advanced) controls may include training, safe handling-riding procedures, talking about risks with horses, better handler-rider match, fit-for-purpose equipment, checking the environment and supervision needs, just to name a few. These safer processes build a more robust risk management program for safer human–horse interactions [42,123].

Higher safety controls may take time to develop and apply. However, when coupled with sufficient handler-rider safety education and communications [124], more positive human safety-first values and beliefs will develop. As a result opportunity is apparent for risk mitigation and reduced horse-related human injures or fatalities as evidenced in ‘high-risk’ industries [125,126,127].

#### 4.3.5. Training Improves Safety Conformance

Training can promote positive human safety behaviours and improve organisational culture [127,128,129,130] although there is often resistance to training interventions. Some experts or more experienced subjects can be harder to engage in training and be willing to learn [131]. This study identified the self-rated more experienced group of respondents were found to be at higher risk, taking less safety precautions. This was a significant quantitative finding for respondents interacting with horses in the survey. Training in safer handling-riding of horses (high-risk activity), increases situational awareness and assists a human in making an informed decision about likely hazards and the risks [132,133].

High-risk workplaces, like mining, construction, aviation and transport implement stringent worker training and supervision programs. A study of an effective evacuation during a fire emergency in road tunnels, concluded safety informed and behavioral trained participants were able to evacuate more reliably than those who did not participant in practical training exercises [134]. The cost-benefit analysis for these industries of ‘value adding’ to workers (especially inexperienced) safety knowledge, behaviour change and risk perceptions is crucial to risk mitigation and often mandatory [135,136,137,138,139,140,141,142].

Equestrians usually want to be successful in horse-related disciplines, irrespective of the financial costs, time invested in horse training, travel, provision of horse welfare needs and even setbacks (e.g., injuries). Horse training typically focuses on training and more training to improve the horse’s performance. However, more recent research is targeting the importance of humans working on their training needs such as, learning more about horse behaviours [143], triggers, herd instincts, safety signals, improved horse handling and safety awareness about the risks of human-harm when interacting with horses [30,144,145,146,147]. This approach makes sense; along with an element of assumption that learning new or safer horse-handling-riding skills, will make it easier to train a horse.

This survey identified all respondents who had participated in any form of horse safety training and leadership continued to expand their knowledge when interacting with horses. Ongoing participation in learning to develop safer human behaviours and practices around horses in variable environments, is likely to promote more frequent safety-first behaviours and choices [148].

However, other elements can influence the success of human training and improved safety knowledge. Some of these variables may include, but are not limited to:

(1) Quality of training methods to produce successful and safe outcomes. Such as, who is providing the training, what is their level of skill and does their knowledge come from a rigid or traditional approach. Alternatively, do they themselves (as coaches) participate in ongoing learning pathways with updated standards [149].
(2) Training usually occurs in a dynamic environment. No one day or training time is the same. Many energy sources can appear unexpectedly, like magpies swooping, loud vehicles, spooks in the arena or a change in weather. All of these unknowns effect the quality of training, the confidence of the human and their ability to maintain focus.
(3) Having a training plan and time to reinforce new training skills is critical to enhance learning retention and improve [150]. Communicating at snail pace (slow and steady) is critical for horsemanship skills to development through extensive practice and time [151]. In equestrian training there are no quick fixes because you are engaging with a sentient independent partner. Finally,
(4) the horse. Our training partner has an independent mind, its own learning style and issues. Each participant in the training session is constantly needing to adapt to each other whereby, assessing and adjusting to presenting risk and situational change. Therefore, to be able to juggle between learning and managing change, a human requires an initial degree of skill and competence.

Likewise, good quality, relevant training [152] delivered in a controlled exposure to environmental variables, is likely to improve safety skills enabling humans to apply more control over hazardous situations. Moreover, increase the likelihood of a human being able to effectively predict, unpredictable horse-related responses [147,153,154,155,156]. We know safety-first training works to reduce unwanted or unplanned injuries, illnesses and fatalities in ‘high-risk’ industries [157,158,159]. Cost–benefit analysis [160] has proven ongoing safety-training in high-risk industries, helps prevent human injuries and fatalities. A similar proactive investment for equestrian would far outweigh significant costs of human rehabilitation [161] and associated legal fees. Moreover, it has the potential to reduce family loss and the devastating impact on social groups and the equestrian industry.

More research is needed to explore what type of training and delivery modes will be successful to encourage equestrians to ‘take-up’ more safety knowledge. In addition, the exploration of other perceived and actual safety barriers existing within equestrian disciplines, may identify ways to overcome human resistance to some proactive safety principles.

### 4.4. Survey Limitations

This study was not completed without some limitations. The survey respondents were predominantly female, which may lead to gender bias [162]. In addition, some of the responses may have been over or underreported based on social desirability bias [163]. Furthermore, the only mode of survey participation was an online format, with no option for telephone, written or face to face completion. All survey responses were subjective with no opportunity for revealed behaviours, observation or discussion. Responses primarily relied on the participant’s self-reported opinion, memory recall, feelings, beliefs and values and was only distributed in the English language [164].

Due to the complexity of language and varying disciplines in equestrian some terminology used may have been misunderstood by respondents, which in fact may limit communication competence [165]. General statements like ‘unsafe behaviours for a horse’ were not defined whereas, other questions included parameters and definitions, which may have been easier for some and not others to interpret. Therefore, survey results may change through improved communication to mitigate any language barriers [166].

Below the “tip of the iceberg” (a metaphor) [167] lies the unknown, assumed and unevaluated risks [168]. This study would be enhanced by further research in the areas of (1) human–horse interactions and risk behaviours, (2) evaluating the benefits of safety training [169] and risk mitigation and (3) the influences of social constructs, culture and tradition and safety-first choices.

## 5. Conclusions

This survey highlighted human (equestrian) beliefs, values and perceptions about risk that have an influence over individual safety behaviors and actions during horse-related interactions, whereby respondents who received income from horse-related activities were more risk tolerant during daily living events, work, and non-work environments. However, there was no consistent relationship between risk-taking and the demographics of respondents’ age or gender.

Survey results have indicated there are many external factors that can motivate a human’s risk-taking predisposition, resulting in either risk-aversion or risk-tolerance. Some proactive (risk-averse) elements to improve safer human–horse interactions include:

(1) quality safety training, coaching and leadership to embed horse handling riding safety first principles;
(2) strict adherence to known (tried and tested) safety practices regardless of skill level or experience; and
(3) the use of higher-level safety controls like, rider–horse match, supervision, environment evaluation and risk assessment, not just personal protective equipment (helmet), the lowest treatment for risk mitigation.

Finally, equestrian as an industry has the opportunity to learn from other high-risk sports and workplaces, about successful risk-mitigation processes to help reduce horse-related human injuries and fatalities. There is no end date for the horse–human relationship. This interspecies connection will survive wars, famine, recessions and politics; it just continues to grow. Therefore, it is critical we learn now how to nurture and safely protect this human–horse relationship, because it adds countless value to society, individuals, and the equestrian industry.

## Figures and Tables

**Table 1:** Respondent’s general risk taking behaviours. Humans have different risk appetites, some are more reluctant to take risks and others more willing in various activities of daily living. This table provides a summary of respondent’s perceptions of risk in non-horse related actives.

	General Risk	Driving	Financial	Leisure-Sport	Occupation	Health	Trust Other
Mean (SD)	4.67 (2.59)	2.54 (2.28)	3.10 (2.32)	4.81 (2.58)	3.77 (2.79)	3.03 (2.37)	3.76 (2.60)
0-Reluctant to take Risk	*n* = 140 (9%)	*n* = 348 (22%)	*n* = 204 (13%)	*n* = 90 (6%)	*n* = 229 (15%)	*n* = 240 (15%)	*n* = 67 (11%)
1	*n* = 84 (5%)	*n* = 313 (20%)	*n* = 72 (17%)	*n* = 118 (7%)	*n* = 209 (13%)	n = 278 (18%)	*n* = 207 (13%)
2	*n* = 124 (8%)	*n* = 256 (16%)	*n* = 262 (17%)	*n* = 133 (8%)	n = 78 (11%)	*n* = 239 (15%)	*n* = 222 (14%)
3	*n* = 158 (10%)	*n* = 179 (11%)	*n* = 223 (14%)	*n* = 154 (10%)	n = 163 (10%)	*n* = 201 (13%)	*n* = 193 (12%)
4	*n* = 142 (9%)	*n* = 118 (7%)	*n* = 149 (9%)	*n* = 158 (10%)	*n* = 140 (9%)	*n* = 165 (10%)	*n* = 144 (9%)
5	*n* = 357 (23%)	*n* = 85 (12%)	*n* = 223 (14%)	*n* = 284 (18%)	*n* = 23 (14%)	*n* = 7 (14%)	*n* = 257 (16%)
6	*n* = 145 (9%)	*n* = 70 (4%)	*n* = 108 (7%)	*n* = 191 (12%)	*n* = 133 (8%)	*n* = 102 (6%)	n = 115 (7%)
7	*n* = 212 (13%)	*n* = 57 (4%)	*n* = 69 (4%)	*n* = 202 (13%)	*n* = 123 (8%)	*n* = 71 (5%)	*n* = 4 (8%)
8	*n* = 132 (8%)	*n* = 34 (2%)	*n* = 36 (2%)	*n* = 147 (9%)	*n* = 99 (6%)	*n* = 29 (2%)	*n* = 85 (5%)
9	*n* = 24 (2%)	*n* = 6 (0%)	*n* = 8 (1%)	*n* = 35 (2%)	*n* = 27 (2%)	*n* = 9 (1%)	*n* = 23 (1%)
10-Prepared to take Risk	*n* = 57 (4)	*n* = 9 (1%)	*n* = 21 (1%)	*n* = 63 (4%)	*n* = 51 (3%)	*n* = 24 (2%)	*n* = 38 (2%)
